# Genome Sequences of Foot-and-Mouth Disease Virus O/ME-SA/Ind-2001 Lineage from Outbreaks in Libya, Saudi Arabia, and Bhutan during 2013

**DOI:** 10.1128/genomeA.00242-14

**Published:** 2014-04-10

**Authors:** Begoña Valdazo-González, Nick J. Knowles, Donald P. King

**Affiliations:** The Pirbright Institute, Pirbright, Woking, Surrey, United Kingdom

## Abstract

The complete genomes of foot-and-mouth disease (FMD) viruses recovered in Libya and Saudi Arabia in 2013 are described here. These viruses belong to an FMD virus lineage (Ind-2001, topotype Middle East-South Asia, serotype O) which is normally endemic in the Indian subcontinent. A contemporary virus sequence from Bhutan is also reported here.

## GENOME ANNOUNCEMENT

Foot-and-mouth disease virus (FMDV) is a single-stranded positive-sense RNA of the genus *Aphthovirus* (family *Picornaviridae*), which spreads rapidly among cloven-hoofed animals, causing important economic losses. FMDV is classified in seven serotypes and multiple genotypes, topotypes, and lineages, from which new FMD viruses can emerge to challenge diagnostic tools and vaccination control programs.

During November 2013, the FAO World Reference Laboratory for FMD (WRLFMD, Pirbright, United Kingdom) received FMDV isolates causing outbreaks in Libya (LIB) and Saudi Arabia (SAU). Phylogenetic analysis of their VP1 coding sequences revealed that these viruses belonged to the Ind-2001 lineage within the Middle East-South Asia (ME-SA) topotype of serotype O (O/ME-SA/Ind-2001), together with contemporary viruses from Bhutan (BHU) and India. These findings were unexpected, since other contemporary FMD viruses from LIB and SAU grouped within the PanAsia-2 lineage (also within the O/ME-SA topotype), or additionally within the East Africa 3 (O/EA-3) topotype in Libya. The O/ME-SA/Ind-2001 lineage was initially identified in the Indian subcontinent in 2001 as a group distinct from the predominant PanAsia lineage (WRLFMD) (1). Since then, Ind-2001 has diverged into four sublineages (a to d) and become the predominant lineage in the region. Outside the Indian subcontinent, only a few sporadic outbreaks have been reported in various countries in the Middle East in 1997, 2001 to 2002, and 2008 to 2009.

Here, we report three representative complete genomes of viruses from the O/ME-SA/Ind-2001 lineage causing outbreaks in BHU, SAU, and LIB during 2013. Viral RNA was extracted from bovine thyroid cell cultures, reverse transcribed, and amplified as previously described (2), but using 20 primer pairs (2, 3) to generate overlapping fragments. The sequences (ABI3730 DNA Analyzer; Applied Biosystems) were assembled, and further analyses were undertaken using SeqMan Pro version 11.2 (Lasergene package; DNAStar, Inc.) and BioEdit version 7.0.5.3 (4). Detailed protocols are available on request.

The genomes are 8,198 to 8,204 nucleotides (nt) in length, including 12 and 14 nt of an artificial poly(C) tract and a poly(A) tail, respectively, and 50 nt derived from primers used for amplification. O/LIB/2/2013 and O/SAU/3/2013 share 99% nucleotide identity with each other and 97% with O/BHU/1/2013. These three viruses differ at 260 sites distributed along the genome. Within the polyprotein, 204 nucleotide substitutions resulted in 45 amino acid (aa) changes within the regions encoding L^pro^, VP1-3, 2C, 3A, 3B^VPg^, and 3D^pol^. Codon 1348 within the viral protein 2C presents a different aa for each of these sequences. These viruses share 93% nucleotide identity with another contemporary virus within the Ind-2001 lineage from Bangladesh, which has a deletion in the S-fragment of the 5′-untranslated region (UTR) of its genome (5). No other complete genomes of members within the Ind-2001 lineage are available in GenBank, and the next closest (92% identity) viruses are within the O/ME-SA/PanAsia-2 lineage from the Middle East and Asia (Myanmar and Bhutan [2004], Israel [2007], and Pakistan [2008]).

The appearance and transboundary spread of O/ME-SA/Ind-2001 reinforce the need to update genetic databases to understand the global epidemiology of FMDV and to develop specific molecular diagnostic tools and vaccines to use in outbreaks due to emerging FMD viruses.

### Nucleotide sequence accession numbers.

The nucleotide sequences for O/BHU/1/2013, O/LIB/2/2013, and O/SAU/3/2013 are deposited in GenBank under accession no. KJ206908 to KJ206910, respectively.
